# Weighted gene co-expression network analysis of nitrogen (N)-responsive genes and the putative role of G-quadruplexes in N use efficiency (NUE) in rice

**DOI:** 10.3389/fpls.2023.1135675

**Published:** 2023-06-07

**Authors:** Narendra Sharma, Bhumika Madan, M. Suhail Khan, Kuljeet S. Sandhu, Nandula Raghuram

**Affiliations:** ^1^ Centre for Sustainable Nitrogen and Nutrient Management, University School of Biotechnology, Guru Gobind Singh Indraprastha University, Dwarka, New Delhi, India; ^2^ Department of Biological Sciences, Indian Institute of Science Education and Research (IISER) - Mohali, Nagar, Punjab, India

**Keywords:** WGCNA (weighted gene co-expression network analyses), G-quadruplexes (G4), NUE (nitrogen use efficiency), epigenetic regulation, hub genes, nitrogen, N-responsive genes, rice

## Abstract

Rice is an important target to improve crop nitrogen (N) use efficiency (NUE), and the identification and shortlisting of the candidate genes are still in progress. We analyzed data from 16 published N-responsive transcriptomes/microarrays to identify, eight datasets that contained the maximum number of 3020 common genes, referred to as N-responsive genes. These include different classes of transcription factors, transporters, miRNA targets, kinases and events of post-translational modifications. A Weighted gene co-expression network analysis (WGCNA) with all the 3020 N-responsive genes revealed 15 co-expression modules and their annotated biological roles. Protein-protein interaction network analysis of the main module revealed the hub genes and their functional annotation revealed their involvement in the ubiquitin process. Further, the occurrences of G-quadruplex sequences were examined, which are known to play important roles in epigenetic regulation but are hitherto unknown in N-response/NUE. Out of the 3020 N-responsive genes studied, 2298 contained G-quadruplex sequences. We compared these N-responsive genes containing G-quadruplex sequences with the 3601 genes we previously identified as NUE-related (for being both N-responsive and yield-associated). This analysis revealed 389 (17%) NUE-related genes containing G-quadruplex sequences. These genes may be involved in the epigenetic regulation of NUE, while the rest of the 83% (1811) genes may regulate NUE through genetic mechanisms and/or other epigenetic means besides G-quadruplexes. A few potentially important genes/processes identified as associated with NUE were experimentally validated in a pair of rice genotypes contrasting for NUE. The results from the WGCNA and G4 sequence analysis of N-responsive genes helped identify and shortlist six genes as candidates to improve NUE. Further, the hitherto unavailable segregation of genetic and epigenetic gene targets could aid in informed interventions through genetic and epigenetic means of crop improvement.

## Introduction

Nitrogen (N) is quantitatively the most important input for crop production after water. However, excessive or imbalanced use of N fertilizers exacerbated by inadequate biological N-fixation or legume-based crop rotation led to poor N use efficiency (NUE). The predominant contribution of N-fertilizers to pollution, biodiversity loss, and climate change made them a global economic and environmental concern (Sutton et al., 2019; Kanter et al., 2020; Raghuram et al., 2021; Sutton et al., 2021; Winiwarter et al., 2022). While agronomic practices and controlled-release fertilizers have been important, crop improvement for NUE is increasingly being advocated at both global (Udvardi et al., 2021) and national levels (Móring et al., 2021). The biological avenues for crop improvement have been extensively reviewed (Long et al., 2015; Mandal et al., 2018; Sinha et al., 2020; Madan et al., 2022; Raghuram et al., 2022).

Rice is the third most produced and consumed crop in the world which feeds half the global population (Norton et al., 2015). It has the lowest NUE among cereals and therefore, accounts for the highest consumption of N-fertilizer among them. Further, its rich germplasm diversity, genomic and functional genomic resources (Kawahara et al., 2013; Li et al., 2014; Chen et al., 2016; Li et al., 2018; Huang et al., 2019) make it an ideal crop to improve NUE (Sharma et al., 2021). Thousands of N-responsive genes have been reported using transcriptome studies in rice (Kumari et al., 2021 and references cited therein), including subspecies *indica* (Pathak et al., 2020) and *japonica* (Mandal et al., 2022). The delineation of the phenotype for N-response and NUE (Sharma et al., 2018; Sharma et al., 2021) enabled its integration with the fast-growing transcriptomic data (Sharma et al., 2021). The development of nutrient-depleted soil (Sharma et al., 2019) enabled N-form-specific studies on nitrate or urea to understand the implications of different N-fertilizers used in developed and developing countries for NUE improvement in rice.

But distinguishing between genes for N-response/NUE and shortlisting the fewest possible target genes for NUE is a work in progress (Kumari et al., 2021; Sharma et al., 2022) that could benefit from newer means for systematic shortlisting. In the meantime, there have been several attempts to validate the candidate genes with varying success (Madan et al., 2022, Raghuram et al., 2022), indicating further scope for identification and shortlisting of more candidates. Important recent progress in this regard has been in the comparative transcriptomics of contrasting NUE genotypes in rice, which projected some potentially important genes (Neeraja et al., 2021; Sharma et al., 2022; Sharma et al., 2023).

Being a quantitative trait, NUE requires the coordinated action of a large number of N-responsive genes contributing to yield. Co-expression network analysis could be an important method to identify coexpressed gene modules but was never employed to understand their roles in NUE or to identify/shortlist candidate gene targets on that basis. Weighted gene co-expression network analysis (WGCNA) is the most popular systems biology tool to identify the modules associated with specific biological processes (Ruprecht et al., 2017; Zhang et al., 2019; Sun et al., 2020). WGCNA has been used to identify key salt-responsive genes in rice (Zhu et al., 2019) and Arabidopsis (Amrine et al., 2015), the potential regulatory mechanism of carotenoid accumulation in chrysanthemum (Lu et al., 2019), receptor-like protein genes involved in broad-spectrum resistance in pepper (Kang et al., 2022) and in the co-expression analysis of rice and maize genes (Chang et al., 2019; Zheng et al., 2019).

There is also growing recognition of epigenetic transcriptional reprogramming in response to nutrients (Séré and Martin, 2020) and epigenetic regulation of N-response through miRNAs (Nischal et al., 2012; Islam et al., 2022) and chromatin remodeling (Li et al., 2021 and references cited therein). An emerging mode of epigenetic regulation that was never explored for NUE involves a special type of non-canonical structure known as a G4 sequence or G-quadruplex. It is produced by Hoogsteen hydrogen bonding in DNA and RNA sequences that contain four short segments of guanine (Burge et al., 2006, Sengupta et al., 2021). While being spread out across the entire genome, these G4 sequences are often abundant in promoter regions, gene UTRs, and telomeres (Griffin and Bass, 2018) It is well recognized that they contribute significantly to chromatin remodelling, gene control, epigenetic regulation, genomic instability and genetic disorders (Shao et al., 2020; Varshney et al., 2020). Despite such significance, the occurrence and roles of G4 DNA and G4 RNA in plant species have not been well studied, barring a few reports on stress (Kopec and Karlowski, 2019). Genome-wide studies of G-quadruplexes have the potential to accelerate progress toward a thorough understanding of their biological implications and practical applications in plants (Cagirici and Sen, 2020; Li et al., 2022). It is therefore of significant interest to investigate the potential of G4 sequences as a fresh method of crop development for NUE.

In the present study, we compiled the largest number of shared N-responsive genes from 8 out of 16 N-responsive transcriptomic datasets in rice and analyzed them by WGCNA. We identified fifteen functional modules of co-expressed genes and the most relevant module for N-response/NUE and the associated biological processes. We validated some genes/processes linked to NUE phenotype and identified novel candidate genes for improving N-response/NUE in rice. We also identified and catalogued sequences of G4 quadruplexes among NUE-related genes and validated their differential expression in contrasting genotypes. A hypothesis/model integrating genetic and epigenetic regulation of NUE has been proposed.

## Materials and methods

### Compilation and annotation of N-responsive genes

To identify N-responsive genes in rice, 16 rice N-responsive microarray datasets available at NCBI GEO were examined. A list of over 18,000 N-responsive genes was compiled from these N-responsive datasets using uniform criteria of Log2FC ≥1, p-value <0.05 with default redundancy removal, as described in Kumari et al. (2021). As very few genes were common to all the N-responsive microarray datasets compiled for this study, individual datasets that were mainly responsible for minimal common genes were eliminated progressively. This led to the shortlisting of eight N-responsive datasets (**Supplementary Table S1**) that had the maximum number of 3020 common N-responsive genes (**Supplementary Table S2**). Gene ontology (GO) enrichment analyses for functional annotation of N-responsive genes were performed Expath 2.0 tool (Chien et al., 2015) using default parameters. The biological processes were obtained using AgriGO v2. (http://systemsbiology.cau.edu.cn/agriGOv2/index.php) and visualized using Heatmapper (http://www.heatmapper.ca).

### Weighted gene co-expression network construction and module identification

In order to independently identify co-expressed N-responsive genes, we performed Weighted Gene Co-expression Network Analysis (WGCNA), using 3020 common genes (**Supplementary Table S2**) from eight N-responsive transcriptomic datasets (**Supplementary Table S1**). We used version 1.69 of the WGCNA software at Bioconductor (http://bioconductor.org/biocLite.R) on the RStudio platform (1.2.5042). The soft threshold method for Pearson correlation analysis of the expression profiles was used to determine the connection strengths and construct a weighted co-expression network among the genes. Average linkage hierarchical clustering was carried out to group the genes based on topological overlap dissimilarity in network connection strengths. To obtain the correct module number and clarify gene interactions, we restricted the minimum gene number to 20 for each module and used a threshold of 0.25. To identify the significant modules related to rice traits, three available experimental criteria in microarray datasets were used as traits including the age of the plant used for tissue sampling, type of tissue (root/shoot/whole plant), and N-treatment. Two approaches were used to identify the significant modules. The first approach used the relationship between the traits and Module eigengenes (MEs), which are the major components for principal component analysis of genes in a module with the same expression profile. The second approach used the relationship between module membership versus gene significance.

### Functional annotation of significant modules

All the genes from each of the modules were analyzed separately using Expath 2.0 (Chien et al., 2015) for their functional annotation by gene ontology (GO) to identify biological, cellular, and molecular processes. In order to study the protein-protein interactions, all the genes from the turquoise module (WGCNA) were used for separate searches for their interacting protein partners on the STRING database version 11 (https://string-db.org/cgi/input.pl?sessionId=Xv9nzTk5s6NX&input_page_show_search=on).

### Data mining for transporters, transcription factors, kinases, and miRNA targets

N-responsive genes encoding transporters were retrieved from the Rice transporters database (https://ricephylogenomics.ucdavis.edu/transporter/), RAP-DB (https://rapdb.dna.affrc.go.jp/), and Transport DB2.0 (http://www.membranetransport.org/transportDB2/index.html). Similarly, N-responsive genes encoding transcription factors (TFs) were retrieved from the databases PlantPAN2 (http://plantpan2.itps.ncku.edu.tw/TF_list_search.php#results), RAP-DB, STIFDB (http://caps.ncbs.res.in/stifdb/), PlantTFDB (http://planttfdb.cbi.pku.edu.cn/index.php?sp=Osj) and Rice Frend (http://ricefrend.dna.affrc.go.jp/multi-guide-gene.html). Kinases were searched by using iTAKdatabase (http://itak.feilab.net/cgi-bin/itak/index.cgi). Plant microRNA database (PMRD- http://bioinformatics.cau.edu.cn/PMRD/) was used for searching the miRNAs that target N-responsive genes.

### Physiological measurements

In order to measure the N-responsive changes in terms of physiological parameters of rice plants, a contrasting pair of rice genotypes namely Nidhi and Panvel1were used for their known low and high NUE respectively (Sharma et al., 2018; Sharma et al., 2021). They were grown in trays filled with nutrient-depleted soil (Sharma et al., 2019) for 21 days. They were fertigated with media (Hoagland and Arnon, 1950) containing urea as the sole source of N at a normal dose of 15mM as control, or a low dose of 1.5mM as a test. These 21 days-old plants were used to measure photosynthesis and transpiration rate using LI-6400XT Portable Photosynthesis System (LI-COR Biosciences, Lincoln, NE, USA). Net photosynthetic rate was measured in terms of CO_2_ assimilated as µ mol CO_2_/m^2^s^1^; transpiration was measured in terms of mol (H_2_O)/m^2^s^1^. Student’s t-test was performed on the test vs. control data. The reference CO_2_ concentration was 410 ± 20 μmol mol^−1^ during the measurements. All LI-COR measurements were carried out at the time of maximal photosynthetic activity between 12:00 noon and 5:00 pm IST. All the measurements were done in at least four replicates.

### G-quadruplex sequences and post-translational modifications

All the gene IDs carrying G4 sequences in exon, promoter, gene, CDS, and UTRs regions were downloaded from the PlantG4DB database (http://ccbb.jnu.ac.in/PlantG4DB/). After combining them and removing redundant gene IDs, they were searched for the N-responsive genes having G4 sequences. To find out the genes associated with post-translational modifications (PTM), all genes associated with PTMs were retrieved from Plant PTM Viewer (https://www.psb.ugent.be/webtools/ptm-viewer/experiment.php), which contains PTM data collected from published reports (Møller and Kristensen, 2006; Nakagami et al., 2010; Xie et al., 2015; He et al., 2016; Qiu et al., 2016; Xiong et al., 2016; Zhang et al., 2016; Hou et al., 2017; Meng et al., 2017; Qiu et al., 2017; Wang et al., 2017; Ying et al., 2017; Mujahid et al., 2018).

### RNA isolation and RT-qPCR analysis

Leaves were harvested from twenty-one days old plants of rice genotypes Nidhi and Panvel1 (contrasting-NUE genotypes) grown in pots with media containing normal or low nitrate levels (15 mM and 1.5 mM potassium and calcium nitrate) and immediately frozen in liquid nitrogen as 100 mg aliquots. Total RNAs were isolated using RNAiso Plus solution and 5 µg each were reverse transcribed using PrimeScript™1st strand cDNA synthesis kit as per the instructions of the supplier (Takara, Japan). Exon spanning primers were designed using the Quant Prime tool (https://quantprime.mpimp577golm.mpg.de/?page=about). RT-qPCR was performed using SYBR Green qPCR MasterMix (GBiosciences, USA) and Aria Mx Real-time PCR System (Agilent technologies, Singapore). The relative abundance of transcripts was calculated by the 2^–△△CT^ method (Livak and Schmittgen, 2001) using actin gene (LOC_Os01g64630) as an internal control. The data were statistically analyzed using GraphPad Prism 6 software. These experiments were performed using two biological and three technical replicates.

## Results

Co-expression analysis of N-responsive genes requires a large enough dataset as well as a large enough number of such datasets. Fortunately, most of the publicly available N-responsive transcriptome datasets (including our own) are available as microarrays, many of which captured thousands of differentially expressed genes. In total, 16 N-responsive microarrays data available at NCBI GEO were used. Venn selections between them revealed that the largest number of 3020 N-responsive DEGs were shared by only 8 transcriptome datasets (**Supplementary Figure 1**). They were used for the rest of the study and their results are described below.

### N-responsive genes associated with NUE-phenotypic traits

**Figure 1:**
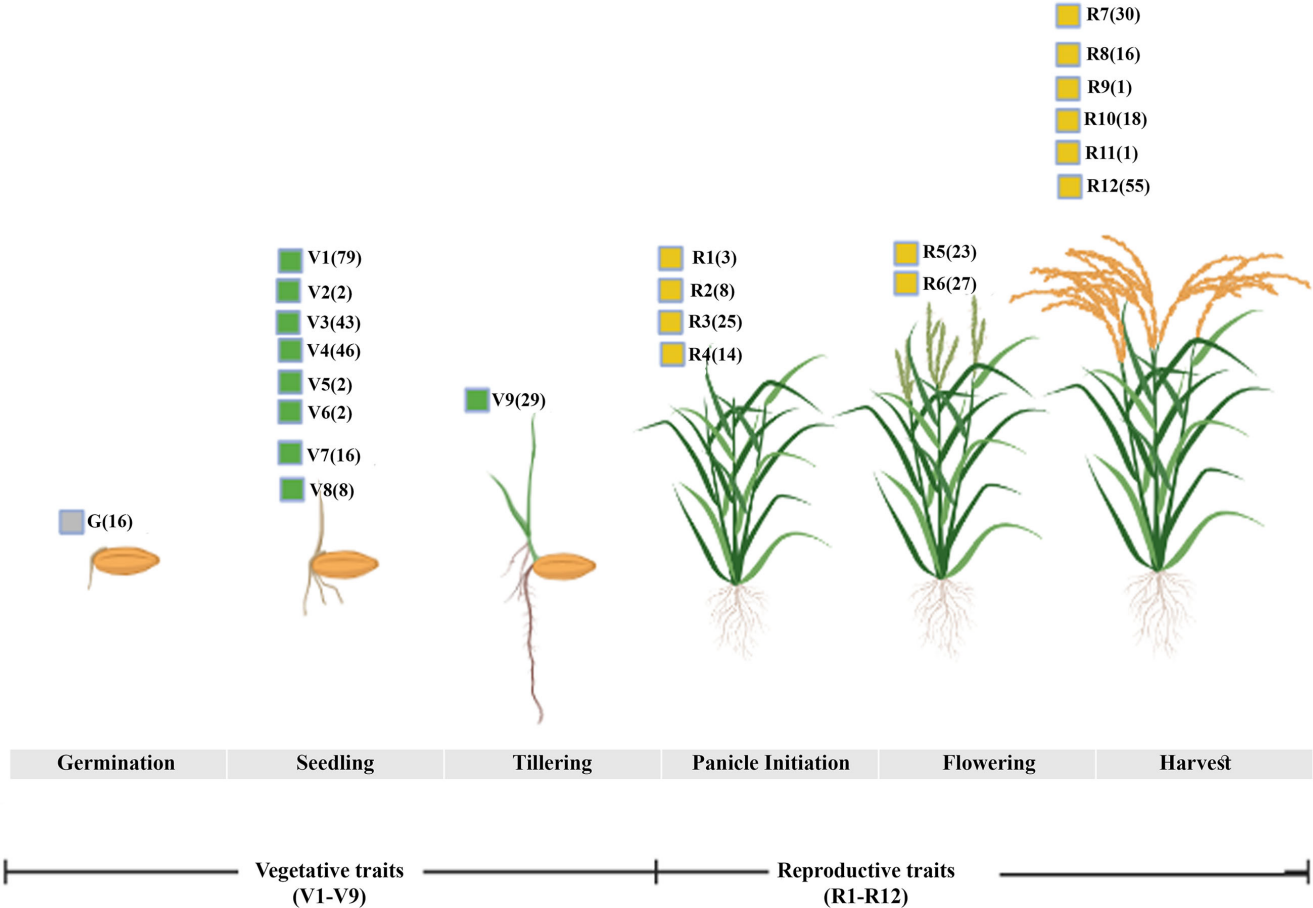
NUE traits checked during various growth phases. The mining of N-responsive genes into the rice database identified genes broadly linked to vegetative traits including germination (G, V1-V9) and reproductive stages (R1-R12) linked to NUE-phenotypic traits. The number of genes associated with each corresponding trait is denoted in the bracket.

In order to find the trait-gene association, we mined for all the traits associated with the 3020 N-responsive genes in the Oryzabase rice database (Kurata and Yamazaki, 2006) yielded 806 genes related to various traits. Among them, 259 genes fell into three phenotypic categories we identified earlier based on lifelong evaluation of 25 traits using six rice genotypes under different N conditions (Sharma et al., 2021). They include133 genes linked to nine vegetative traits (V1-V9), 110 genes linked to 12 reproductive traits (R1-R12), and 16 genes linked to germination (G) rate and ratio (**Figure 1**; **Supplementary Table S3**). Six genes across these three categories were associated with all the eight phenotypic traits that we identified earlier for NUE (Sharma et al., 2021). These traits were, germination, flowering, shoot length, fresh and dry biomass, root length, chlorophyll content and total plant height. The six genes associated with all these 8 NUE traits were, Os06g0603000 (Photoperiod-sensitivity-5), Os07g0497100 (Chromatin Remodeling 4), Os08g0162100 (ABERRANT SPIKELET AND PANICLE1), Os04g0498600 (S-adenosylmethionine decarboxylase), Os08g0127100 (Lysine/Histidine transporter 1), and Os03g0669200 (heterotrimeric G protein beta 1 subunit, RGB1). They could be tested on priority for nitrogen use efficiency.

### Biological pathways and sub-cellular locations of genes involved in N-response/NUE

**Figure 2:**
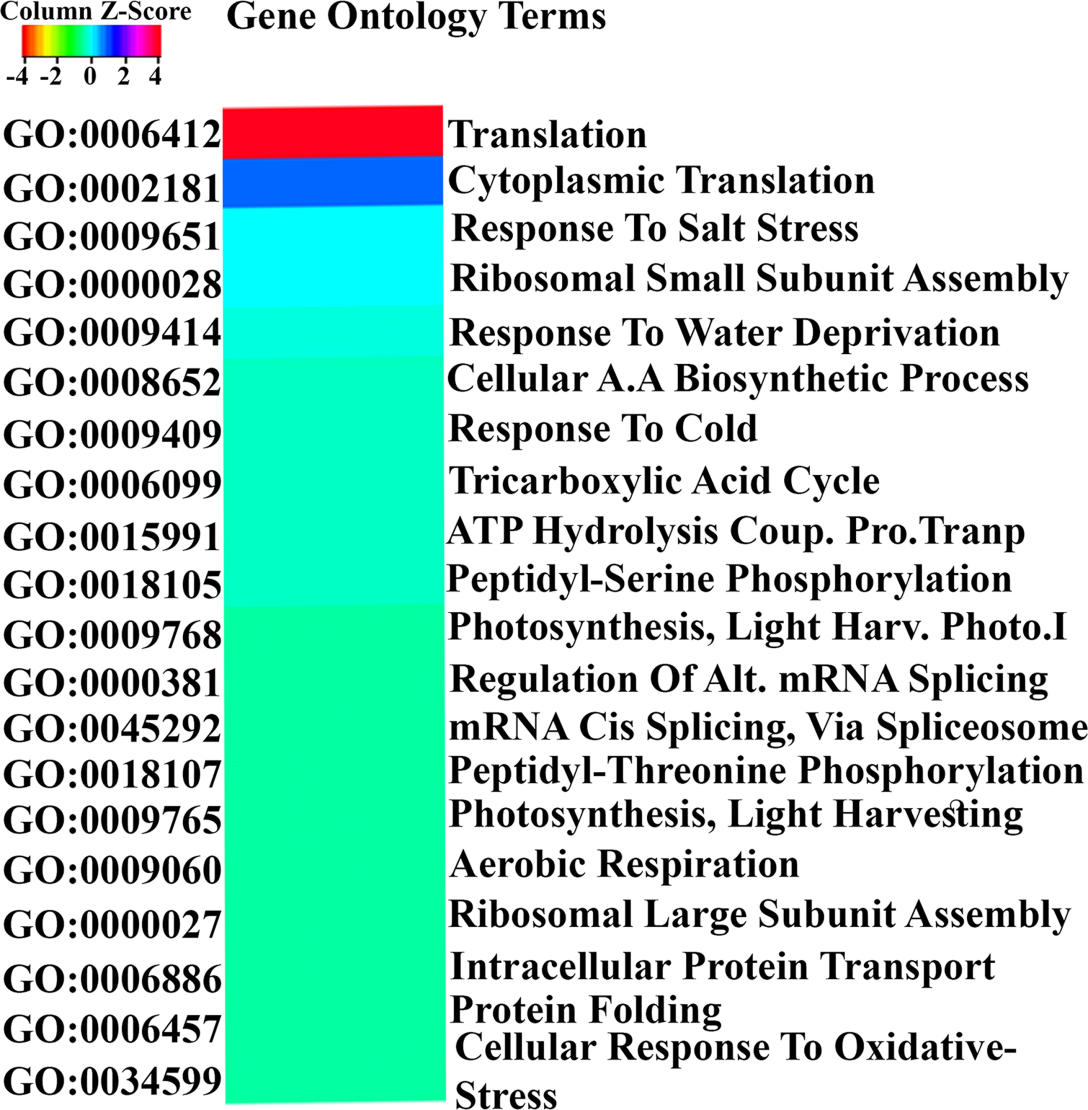
Gene Ontology analysis of N-responsive genes. GO analysis of the N-responsive genes performed using Expath for biological processes based on P values and FDR. The top 20 statistically significant biological processes (P < 0.05) were visualized using Heatmapper.

Gene Ontology (GO) analysis of all 3020 N-responsive genes using EXPath 2.0 for biological processes based on P values and FDR showed the involvement of translation, salt stress, water deprivation, amino acid biosynthetic process, cold stress, tricarboxylic acid cycle, peptidyl-serine phosphorylation, photosynthesis, mRNA splicing and respiration, among others. The details of GO-enrichment analyses are provided in **Supplementary Table S4**. The top 20 statistically significant biological processes (P < 0.05) were visualized using Heatmapper (**Figure 2**). Similar processes were also obtained when GO analysis was carried out using AgriGO v2. They include translation, carbohydrate metabolism, hormone, and photosynthesis (**Supplementary Table S5**). Many DEGs were also mapped to calcium metabolism, amino acid metabolism, ubiquitination, and tricarboxylic acid cycle among others, suggesting crosstalk between these pathways.

### Co-expression network analysis reveals coregulated modules of N-responsive genes

**Figure 3:**
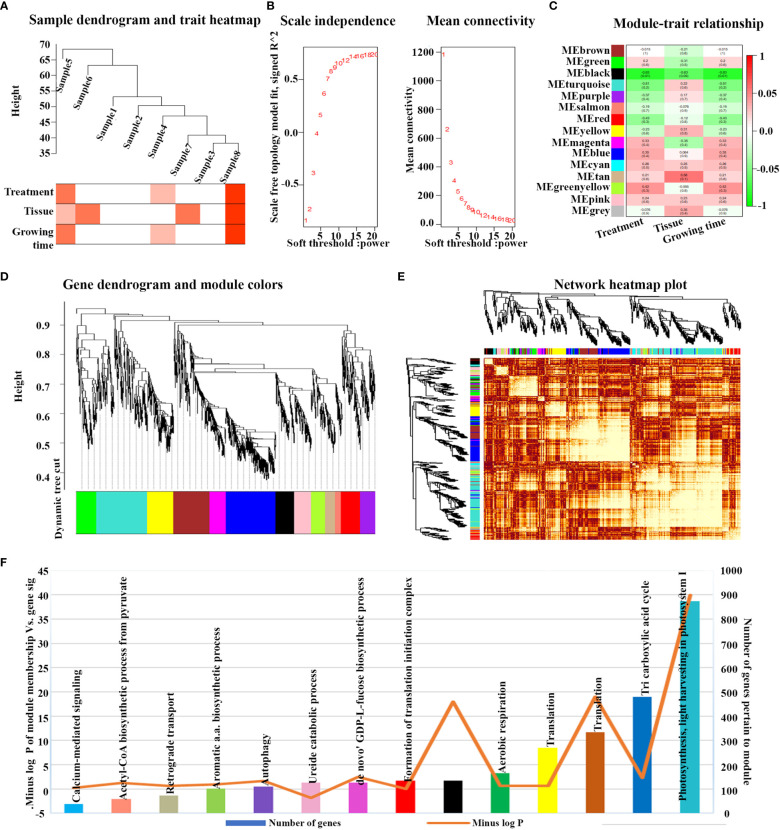
Weighted gene co-expression analysis (WGCNA) and module’s associated processes of N-responsive genes. **(A)** All eight N-responsive datasets passed the cutoff thresholds, and the sample dendrogram as well as the corresponding traits were deemed suitable for network analysis. **(B)** Topology of the network analyzed for various soft-thresholding powers. On the x-axis, the weight parameter β is represented, while the left figure’s y-axis represents the correlation coefficient squared between log(k) and log(p(k)) in the corresponding network. On the right graph’s y-axis, the mean of all gene adjacency functions in the corresponding gene module is represented. **(C)** Matrix showing Module-Trait Relationships (MTRs) of 15 different modules under different conditions. The y-axis denotes the module names, while the x-axis denotes the conditions. The numbers in the table correspond to the Pearson correlation coefficients, and the color legend is used to show the correlation level. The heatmap’s right side displays the correlation’s intensity and direction, with red indicating a positive correlation and green indicating a negative correlation. **(D)** A hierarchical cluster tree of the common genes displaying coexpression modules is shown. The assigned modules are depicted by branches and color bands, while the major tree branches are labeled in distinct colors. Genes are represented by the tips of the branches. **(E)** The interaction between co-expression based on eigengenes as indicative patterns and evaluated the similarity of each module by correlating their respective eigengenes and cluster dendrogram is displayed. The axes’ colors indicate their respective modules, and the heatmap’s yellow intensity represents the degree of overlap, with darker yellow denoting greater overlap. Indicative patterns and evaluated the similarity of each module by correlating their respective eigengenes. **(F)** Showing the turquoise module, which includes 29% of the genes, had the largest number of genes.

Out of the 16 N-responsive microarray datasets considered, only eight of them had the maximum number of shared N-responsive genes (3020) and only these 3020 genes were used for the WGCNA analysis. Prior to analysis, it was verified that the sample dendrogram and corresponding traits of all the 3020 N-responsive genes passed the cutoff thresholds and were suitable for network analysis (**Figure 3A**). As the soft threshold power value is a critical parameter that affects the independence and average connectivity degree of the co-expression modules, β = 6 was selected with scale-free R^2 = ^0.928 for the analysis, based on prior screening for network topology (**Figure 3B**). The gene co-expression network was constructed using hierarchical clustering of the calculated dissimilarities resulting in fifteen different modules (**Figures 3C, D**; **Supplementary Table S6**). We employed eigengenes as indicative patterns and evaluated the similarity of each module by correlating their respective eigengenes (**Figure 3E**). The turquoise module, which encompasses 29% of the genes, had the highest count of genes with a total of 875 (**Figure 3F**). The blue module had the next largest number with 479 genes, followed by the brown module with 334 genes, yellow with 270, green with 165, black with 133, and so on.

### Significant modules reveal the enrichment of photosynthesis and other processes

To identify the physiological processes involved in N-response/NUE, we examined the correlation between genes from the significant co-expression modules with their process annotations. The identification of the modules was based on two criteria: a module-trait relationship with an R2 value greater than 0.5 and a significant p-value for the relationship between module membership and gene significance. Two modules, namely black and turquoise, were identified based on these criteria (**Figure 3C**). Nevertheless, the turquoise module demonstrated a higher score than any other module according to the second criterion, suggesting its stronger correlation with the NUE traits (**Figure 3F**). Both modules exhibited a negative correlation with the treatment and age of the plant from which the tissue was sampled. However, a positive correlation was observed in the turquoise module with respect to the type of tissue sampled. These findings suggest that the spatial co-regulation of gene expression in different tissues/organs of the plant may be more significant in N-response/NUE than temporal co-regulation in terms of different stages of development.

Gene ontology analysis of all the modules revealed their involvement in various processes (**Supplementary Tables S4, S5**), as well as their significance in each module (**Figure 3F**). It revealed photosynthesis as the most significant process in the turquoise module, TCA cycle in the blue module, translation in the brown/yellow module, respiration in the green module and other physiological processes including proteolysis and defense in the black module (**Supplementary Table S7**). Other significant processes include jasmonic acid mediated signaling pathway, seed coat development, glutamate metabolic process, response to cold, D-xylose metabolic process, cellular amino acid metabolic process.

### PPI network of co-expressed N-responsive genes reveals hub genes

**Figure 4:**
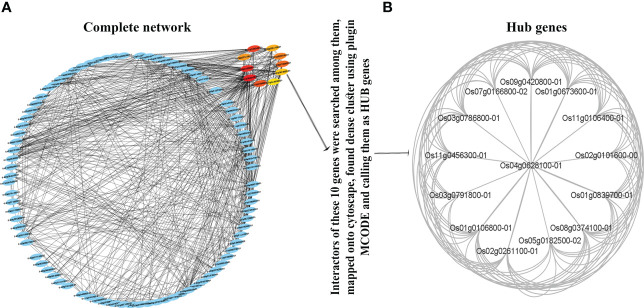
Protein-protein interaction (PPI) network and Hub Genes. Network based on functional annotation of the co-expressed genes from the turquoise module. The interactors were identified using STRING database and visualized by Cytoscape based highly significant PPI enrichment score (1.0e-16). **(A)** Interaction modules involved in the associated processes; **(B)** The highly connected genes called as Hub Genes among the interactors of the top 10 genes.

In view of better functional annotation of the co-expressed genes from the turquoise module, this module was chosen for protein-protein interaction (PPI) network analysis. All the interacting partners of the turquoise module were retrieved from the STRING database (Szklarczyk et al., 2015, **Supplementary Table S8**). They were found to have a highly significant PPI enrichment score (1.0e-16). They were ranked by the experimentally validated protein–protein interaction score and their networks with 518 nodes and 3104 edges were visualized by Cytoscape version 3.91 (**Figure 4A**) to reveal the interaction modules involved in the associated processes (**Supplementary Table S7**). Gene Ontology was performed using ExPath2.0 to find out the processes aided by such protein interactions (**Supplementary Table S8**). It revealed photosynthesis, transport, protein-chromophore linkage, glycolytic process, sterol biosynthetic process, mRNA splicing, chromatin organization and oxidation-reduction among others that need interaction between products of co-expressed N-responsive genes (**Supplementary Table S8**).

To select the hub genes from the network, firstly CytoHubba plugin was used with the MCC algorithm, which provided the top 10 genes with default parameters. Secondly, the MCODE plugin was used to identify the x highly connected genes among the interactors of these top 10 genes. The CYTOHUBBA and MCODE plugins only provide statistically significant genes or gene clusters by default. Therefore, these x highly connected genes qualify to be called as hub genes (**Figure 4B**). Their functional annotation revealed their involvement in the ubiquitin process.

### Transcription factors and transporters coordinate N-response/NUE

**Figure 5:**
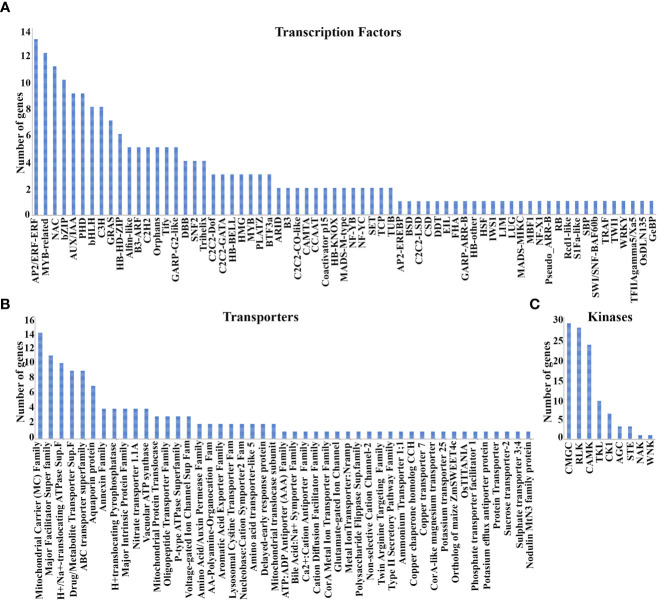
Transcription factors, Transporters, and Kinases associated with NUE. **(A)** 67 classes of TFs encoded by 210 N-responsive genes; **(B)** 15 Major and 32 minor transporter gene families identified in 92 and 45 N-responsive genes respectively; **(C)** Nine kinase families identified in 98 N-responsive genes.

N-response spans thousands of genes and a fraction of those that are additionally associated with yield contribute to NUE as a multi-genic trait (Kumari et al., 2021; Sharma et al., 2021). Identification of the most contributing genes has been a challenge and could be aided by shortlisting them from the functional groups emerging from co-expression analyses, such as transcription factors (TFs), transporters, etc. We searched for the 3020 N-responsive genes among different TF databases and identified 67 classes of TFs encoded by 210 N-responsive genes. They include 26 major classes (≥3 genes) totaling 156 genes and 41 minor classes (¾2 genes) totaling 54 genes (**Supplementary Table S9**). They are AP2/ERF-ERF, MYB-related, NAC, bZIP, AUX/IAA, PHD, bHLH, C3H, and GRAS among others (**Figure 5A**; **Supplementary Table S9**). Among the co-expressed modules, the turquoise module was predominant for transcription factors (55) followed by blue (33), yellow (22), brown (20), pink (16), red (14), and nine others. Gene counts based on Venn analysis of these 210 TFs with predicted NUE-related TFs (Kumari et al., 2021) confirmed 32 of them. Only one of them (Dof1) was previously reported as associated with NUE (Kurai et al., 2011), but our analysis offers many more (31) TFs as candidates to improve N-response/NUE.

N-transporters are important regulators of source-sink dynamics (Tegeder and Masclaux-Daubresse, 2018) and some were indeed associated with NUE (Masclaux-Daubresse et al., 2010; Wang et al., 2018; Wang et al., 2019; Hou et al., 2021, Nazish et al., 2021). We searched for the 3020 N-responsive genes among Rice transporter DB, RAP DB, Transport DB2, and identified 15 major transporter’s families (≥3 genes) totaling 92 genes and 32 (≤2 genes) minor transporters families totaling 45 genes (**Supplementary Table S10**). The top 5 transporter’s families are the mitochondrial carrier (MC) family, major facilitator superfamily (MFS), H+- or Na+-translocating F-type, V-type and A-type ATPase (F-ATPase) superfamily, drug/metabolite transporter (DMT) superfamily, ABC transporter superfamily (**Figure 5B**). Their detailed description has been provided in **Supplementary Table S10**. Among the coexpressed modules, the turquoise module has maximum transporters followed by blue, brown, pink, black, and others. A Venn analysis comparing these 132 transporters to earlier predicted NUE transporters confirms 17 of them as NUE transporters. Out of the analyzed transporters, AMT1.1 and NRT1.1 were validated in the field for NUE. Thus, our analysis identified several other transporters as potential candidates for improving N-response/NUE.

### Protein kinases in N-response/NUE

Protein kinases are known to play an important role in N-response and NUE in crops (Fataftah et al., 2018; Hsieh et al., 2018; Jiang et al., 2018; Perchlik and Tegeder, 2018; Xiong et al., 2019). Here, we identified 98 N-responsive genes encoding 9 kinase families (**Figure 5C**; **Supplementary Table S11**) using the database iTak (http://itak.feilab.net/cgi-bin/itak/index.cgi). They are glycogen synthase kinase (GSK) and CDC-like kinase (CLK; CMGC: 27), receptor-like kinases (RLK: 26), Ca2+/calmodulin-dependent protein kinases (CAMK: 22), Tyrosine kinase-like (TKL: 9), casein kinase 1(CK1: 6), AGC (3), serine/threonine protein kinase (STE: 3), a numb-associated family of protein kinases (NAK: 1) and with-no-lysine protein kinases (WNK: 1). Among the coexpressed modules, the turquoise module was predominant for kinases (25) followed by blue (15), brown (11), yellow (10), green (8) and nine others. Venn analysis of these 98 kinases with the previously predicted NUE-related kinases in rice (Kumari et al., 2021) enabled shortlisting of 18 of them as potentially critical for NUE. Among them, four kinases namely GUDK, OsBSK3, OSK1, and OSK3 were previously field-validated for yield but not for NUE. Thus, our analysis offers a shortlist of kinases as candidates to improve N-responsive yield and therefore NUE.

### miRNAs in N-response/NUE

To understand the role of miRNAs in post-transcriptional regulation of N-response/NUE, targets for miRNAs were searched among the 3020 N-responsive genes using the Plant miRNA database. The search identified 67 unique miRNA targets. The details of their genes and functions along with references are provided in **Supplementary Table S12**. Their gene ontology analysis by ExPath2.0 revealed the GO terms such as pollen development, splicing, and RNA processing among others (**Supplementary Table S12**). This indicates the role of these miRNA targets in regulating yield through RNA splicing. Among the coexpressed modules, the turquoise module was predominant for these miRNA targets (15) followed by blue (14), brown (10), magenta (6), and eight others. Venn analysis of these 67 miRNA targets with the previously predicted NUE-related miRNA targets (Kumari et al., 2021) enabled shortlisting of 4 of them as potentially related to NUE. They are, osa-miR1424, osa-miR170a, osa-miR1848 and osa-miR1861. These identified microRNAs have previously been reported to play a role in regulating rice grain development (Lu et al., 2008; Zhu et al., 2008) but not in NUE. Thus, our analysis offers a shortlist of miRNA targets as candidates to improve NUE.

### N-regulated post-translational modifications in rice

Initial gene ontology analysis of N-responsive DEGs in Nidhi revealed many terms associated with post-translational modifications (PTM) such as phosphorylation, de-phosphorylation, hydrolase activity, glycosylation, and ubiquitination (**Supplementary Table S13**). In order to find N-responsive DEG-encoded proteins that can be modified post-translationally, the 3020 N-responsive genes were searched in the PTM viewer database (https://www.psb.ugent.be/webtools/ptm-viewer/experiment.php). We found 1918 gene IDs in the entire WGCNA data, of which the maximum number of PTMs (1056) were found for Lysine 2-Hydroxyisobutyrylation followed by Phosphorylation (651), Lysine Acetylation (102), Carbonylation (71), Ubiquitination (20), N-glycosylation (12), Succinylation (4) and Malonylation (2) (**Supplementary Table S13**). Gene counts using Venn analyses between these PTM genes and previously predicted NUE-related transcription factor genes in rice (Kumari et al., 2021) shortlisted16 DEGs encoding post-translationally modulated TFsfor NUE. Out of the 16 TFs, 14 (ASD1, DOS, OsbZIP12, OsNAC6, OsHAM2, OsPRR73, OsBIHD1, OsSPL9, OsHDT1, OsABF3, OsARF10, OsFBH1, OsNTL5 and OsMYBS2) underwent post-translational modification by phosphorylation, while the remaining two (OsC3H33 and OsCOL4) were modified by acetylation (**Supplementary Table S13**).

Similarly, 11 transporters with post-translational modifications were also found. Of these, six (OsLAX1, AMT1.1, OsEIN2, OPT, OsNPF2.4 and OsSUT2(t)) were modified via phosphorylation, while OsPAPST1 and OsABCC1 were modified by 2-Hydroxyisobutyrylation. Additionally, OsBT1-3 and OsABCC13 were modified via acetylation, while OsHT was modified through ubiquitination (**Supplementary Table S13**). Among the coexpressed modules, the turquoise module was predominant for these PTMs (550) followed by blue (304), brown (233), yellow (169), green (113) and ten others (**Supplementary Table S13**). Notably, out of these all TFs and transporters identified in this analysis, only one transporter namely AMT1.1 was field-validated for NUE in rice (Ranathunge et al., 2014). This analysis offers many more post-translationally regulated N-responsive genes involved in NUE from coexpression modules.

### G-quadruplex sequences could epigenetically regulate N-responsive yield and NUE

To determine the presence of G4 sequences in N-responsive genes, we obtained the complete Oryza sativa G4 sequence data from PlantG4DB. After removing duplicate genes, we identified unique gene IDs that contained G4 sequences and performed Venn Selection with our 3020 N-responsive genes to identify 2298 genes. They were found to be distributed on all 12 chromosomes, though chromosomes 1, 2, and 3 accounted for over 50% of them. A detailed search for G4Q subclasses revealed that 2065 genes contained them in their mRNA/gene region, 1977 in their exons, 1649 in their CDS, 716 in 5’UTRs, 399 in promoters, and 161 in 3’UTR regions (**Supplementary Table S14**). Their statistical significance was confirmed by Fischer’s exact test and the details are provided in **Supplementary Table S15**. We also found a 17.6% higher occurrence of G4s in the plus/antisense strand compared to the negative/sense strand.

Among the identified WGCNA modules, 674 genes of the turquoise module were found to have G4 sequences followed by blue, brown, and other modules. Their details are presented in **Supplementary Table S16**. Gene ontology analysis of these N-responsive genes having G4 sequences revealed that they were involved in carbohydrate metabolism, nitrogen transport, signaling, respiration, and water deprivation among others (**Supplementary Table S17**). As yield association is an important differentiator in N-response and NUE (Sharma et al., 2021), we used a list of 3532 yield related genes compiled from journal literature and online databases as described earlier (Kumari et al., 2021). Their Venn selection with the 2298 genes having G4 sequences revealed 389 genes as both N-responsive and yield associated and therefore NUE-related (**Supplementary Table S18**). To our knowledge, this is the first shortlisting of G4s genes as important candidates for epigenetic improvement of NUE.

To confirm the differential N-responsiveness of some of these shortlisted genes, we selected 18 NUE-related genes (N-responsive and yield-associated genes) containing different location categories of G4 sequences (5’UTR, 3’UTR, cds, exon, mRNA and promoter) for further validation by RT-qPCR. The list of primers used in this study is provided in **Supplementary Table S19**. As negative controls, we used a non-N responsive gene without G4 sequences (Os01g0940000) and a non-N responsive gene with G4 sequences (Os09g0456200). Their expression was nearly unaltered, whether in terms of genotypes or N-treatments (**Supplementary Figure 2**). In addition, actin was used as an internal housekeeping control for RT-qPCR to test low nitrate response against normal nitrate. Relative to these controls, the expression of 7 test genes was validated for differential expression, either in terms of genotype or nitrate response (**Supplementary Figure 2**). Interestingly, 4 out of these 7 genes showed contrasting patterns of N-response between contrasting genotypes, while the other 3 showed similar up or downregulation by nitrate in both the genotypes. The genes that showed contrasting patterns were, *OsCYP20-2* (Os05g0103200), *OsGLP2-1* (Os02g0532500), *TubA2* (Os11g0247300) and *OsDXS2* (Os06g0142900), while the other three genes namely *SPDT* (Os06g0143700), OsASNase2 (Os04g0650700) and OsNRT1.1A (Os08g0155400) showed a similar pattern of regulation. These differences in regulation could be of particular interest to further dissect the mechanism of regulation of NUE, or to validate their potential for crop improvement.

### G-quadruplex sequences differentiate genes involved in N-response and NUE

G-quadruplex sequences are known in genes that are associated with energy homeostasis, oxidative stress, and signaling pathways such as AMP kinases and TOR kinases (Xu et al., 2010; Robaglia et al., 2012; Dobrenel et al., 2013). TOR kinases have their role in the development of leaf and shoot via the GTPase ROP2 in response to nitrogen (Tulin et al., 2021). Therefore, we propose that nitrogen-responsive genes are important targets for the formation of G-quadruplex sequences and are regulated based on external N-availability. Gene ontology analysis of the N-responsive genes containing G4 sequences reveals their involvement in carbohydrate metabolism, water deprivation, nitrogen transport, respiration, among others (**Supplementary Table S17**). Interestingly, their Venn selection with the previously reported NUE-related genes revealed that 17% of the N-responsive genes containing G4 sequences are related to yield therefore, NUE. This provides the first ever estimate that upto 17% of the N-responsive genes could participate in NUE through epigenetic regulation mediated by G-quadruplex sequences, subject to further validation. Thus, G4 sequences could provide an effective means for differentiating between N-response and NUE at the gene level. The remaining 83% of them could either use genetic mode of regulation or other forms of epigenetic regulation besides G4Q.

### NUE involves better photosynthesis, transpiration, and seed germination in low urea

**Figure 6:**
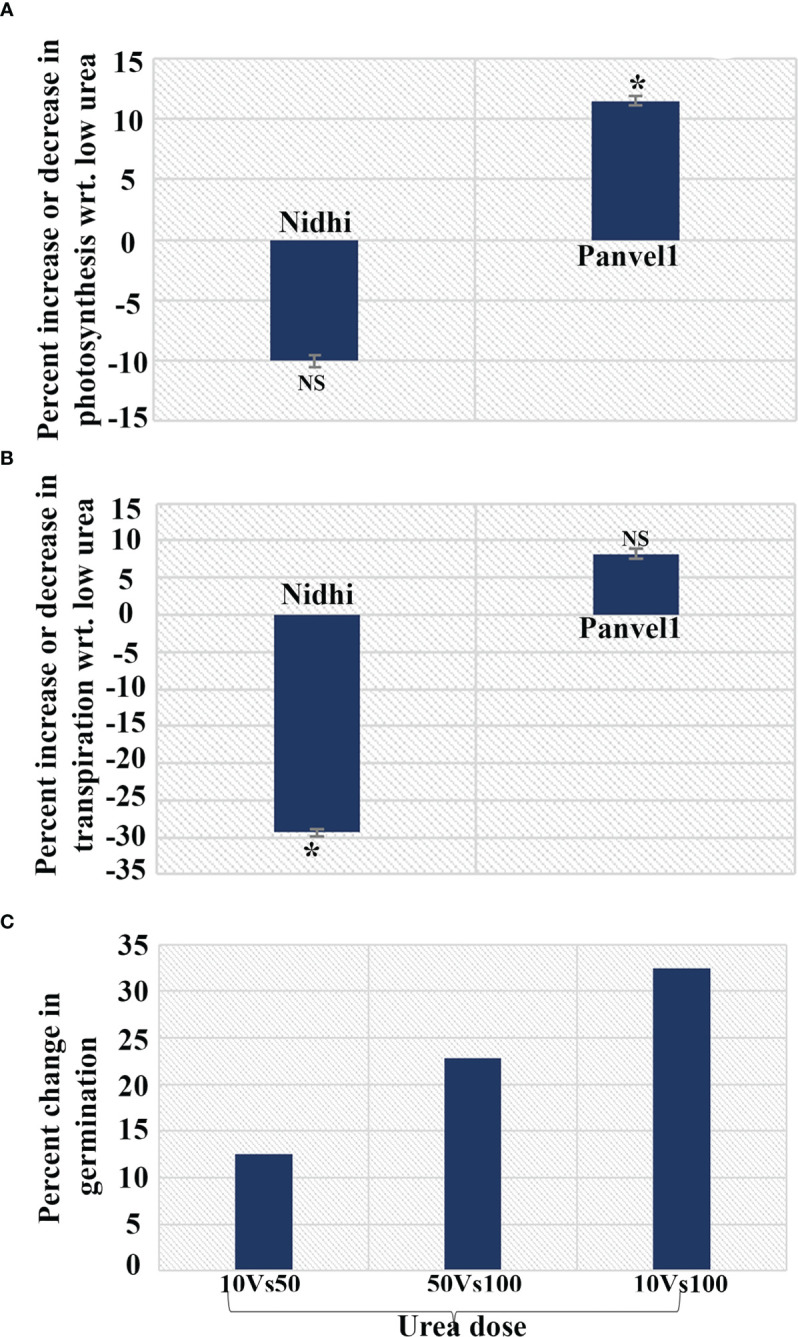
N-responsive changes in physiological parameters in a contrasting pair of rice genotypes. Changes measured in **(A)** Photosynthesis; **(B)** Transpiration and **(C)** Germination under low urea and normal conditions in Nidhi (low NUE) and Panvel1 (high NUE) rice genotypes. The test of significance (P < 0.05) has been shown as the star, while NS represents non-significance.

From the gene ontology (GO) analysis of N-responsive genes containing G4s genes constituting the turquoise co-expression module, photosynthesis transpiration and seed germination were chosen for physiological validation by LICOR, while the genes tested above by RT-qPCR span other processes such as metabolic and abiotic stress processes, apart from chlorophyll and photosynthesis. Using 21 days old potted rice plants, photosynthesis and transpiration were measured on a pair of contrasting rice genotypes namely, Nidhi (low NUE) and Panvel1 (high NUE) using LICOR6400XT as described in materials and methods. Photosynthesis was significantly higher (P < 0.05) in low urea (1.5 mM N) over normal urea (15 mM N) for the high NUE genotype Panvel1, while it was lesser in the case of Nidhi (**Figure 6A**). A similar pattern was also observed for transpiration, though not found to be statistically significant (**Figure 6B**). In an independent experiment to test N-responsive germination in another high NUE genotype of rice (Vikramarya), surface sterilized and presoaked seeds were grown in Petri plates on moist cotton containing Arnon Hoagland medium (Sharma et al., 2018). The media contained either normal Ndose given as urea (15mM), 50% of normal N (7.5mM), or 10% N (1.5mM). By counting the visibly germinated seeds, it emerged that the highest % germination was found in 10% of normal urea (1.5 mM), followed by 50% N and 100%N (**Figure 6C**).

## Discussion

Several transcriptomic datasets of N-responsive genes are now available in various crops including rice, and they contain a vast amount of valuable information that has yet to be fully utilized. They include the underlying processes, shortlisting of candidate genes, identification of QTLs, miRNAs and their targets (Kumari et al., 2021). Some of them are specific to different sub-species of rice, such as *indica* (Pathak et al., 2020; Sharma et al., 2022), *japonica* (Mandal et al., 2022, others) or different sources of N such as nitrate, ammonium, or urea (Sharma et al., 2021). But together, they span diverse genotypes, N-forms and growth conditions yielding thousands of DEGs and enabling comprehensive meta-analyses. Identification of co-expressed genes/modules by methods such as WGCNA is one of the ways to distill the essence from all these datasets, but so far this was done only with individual N-responsive transcriptomic datasets in rice (Coneva et al., 2014; Zhang et al., 2019; Ueda et al., 2020; Yang et al., 2020b). Therefore, the present study utilized 16 microarray datasets for which the DEGs could be extracted from publicly available datasets and shortlisted 8 of them that shared the largest number of 3020 DEGs for WGCNA and other analyses.

Even though several RNAseq transcriptomes have also been published (SRP253184), very few of them revealed their DEGs and their small number of a few hundred DEGs did not meet the criteria for WGCNA and hence not considered for this study. Our analyses of the 3020 N-responsive DEGs common to 8 transcriptomes included WGCNA for genetic regulation and G-quadruplex sequences and miRNAs for epigenetic regulation to quantify their relative contribution to the NUE trait and to propose a model for its regulation.

Genes with similar expression patterns may participate in similar biological processes or networks. Further, positively coexpressed genes within the same pathway tend to cluster in close proximity within the pathway structure, whereas negatively correlated genes generally occupy more distant positions (Mao et al., 2009). Similar results were found in plants when coexpression networks of 1,330 genes derived from the AraCyc metabolic pathway database of *Arabidopsis thaliana* were analyzed (Wei et al., 2006). Our WGCNA of 3020 N-responsive genes shared by 8 N-responsive microarray datasets yielded 15 modules. The turquoise module had the largest number of 875 genes with the highest significance and proportion of functional categories. Of these, 34% were transporters, 26% TFs, >22% miRNAs, 25.5% kinases, >28% PTMs, and>29% G4 sequences. This module contains the largest number of predicted NUE-related genes by Kumari et al. (2021), apart from the field-validated ammonium transporter and nitrate transporter (OsNPF2.4, Fan et al., 2016). This makes the turquoise module the most suitable one to identify hub genes and important processes for NUE. They include photosynthesis, water transport, and seed germination, which we had earlier shown as important processes for N-response/NUE (Sharma et al., 2018; Kumari et al., 2021; Sharma et al., 2021; Mandal et al., 2022; Sharma et al., 2022).

Gene ontology analyses revealed that biosynthesis and transport of nitrogen, photosynthesis, water deprivation, translation, signal transduction, respiration and peptide biosynthetic process were prominent biological classes of N-responsive genes (**Supplementary Table S4**; **Figure 2**), suggesting the role of nitrogen-responsive genes in respiration, photosynthesis, and water deprivation, etc. These findings extend our experimental observations in *indica* (Sharma et al., 2018; Sharma et al., 2022) Kumari et al. (2021) and *japonica* rice (Mandal et al., 2022), which also indicated the importance of some transcription factors. In this study, we found 210 DEGs encoding transcription factors falling into 67 categories (**Supplementary Table S7**). A few genes associated with NAC, MYB and GRASS are among the top categories, while DOF and MADS are among the bottom and have been implicated earlier in yield or NUE (Madan et al., 2022). Among them, DOF1 is well-known to improve NUE (Yanagisawa et al., 2004) and ARF4 has been reported to improve yield (Hu et al., 2018). Therefore, it may be attractive to validate the remaining TFs reported here for their role in NUE.

Nitrogen-responsive transporters can uptake either nitrate or ammonium ions, amino acids, or urea through their respective families of transporters for plant growth, development, yield, and NUE (Kumari et al., 2021; Madan et al., 2022; Sharma et al., 2022). In this study, the 132 transporters encoded by urea-responsive genes include 17 that we previously predicted to be involved in NUE (Kumari et al., 2021; **Supplementary Table S8**). Interestingly, the mitochondrial carrier family tops among the other transporter families and their further characterization, and shortlisting might reveal new targets for NUE.

A common limitation of transcriptomic analyses is that they do not adequately account for the role of post-translational modifications (PTMs) in the response to environmental signals despite their importance in signal transduction. They have also not been explored in N-response till recently (Pei et al., 2019; Wang et al., 2021; Han et al., 2022) and NUE (Sharma et al., 2022). Our bioinformatic analysis revealed 8 types of N-responsive PTMs of 1918 proteins, of which Lysine 2-Hydroxyisobutyrylationemerged as most predominant, followed by phosphorylation for their potential role in N-response/NUE (**Supplementary Table S12**). Phosphorylation has recently been acknowledged to be a crucial PTM for N response (Han et al., 2022) and NUE (Sharma et al., 2022). Here, we report that the overall targets of PTM in N-response/NUE include 14 transcription factors (**Supplementary Table S13**) and 6 transporters (**Supplementary Table S14**).

G4s are considered to act as molecular switches to regulate gene expression in metazoan cells (Eddy and Maizels, 2006). In plants, a few studies have reported the role of G4 sequences, such as in transport and gene expression (Garg et al., 2016), growth and development (Yang et al., 2020b), hydrolase activity (Cagirici et al., 2021), stress response, energy status, and sugar availability (Yadav et al., 2017). Here, we report their role in N-response or NUE for the first time. We found the highest occurrence of G4Q in the turquoise module of N-responsive DEGs in rice. The occurrence of G4s in their mRNAs, exons and CDS suggests their role in the regulation of gene expression (Andorf et al., 2014), while their occurrence in UTRs suggests their role in post-transcriptional regulation (Wang et al., 2015). Among the subtypes of G4s, we found that G2 sequences were >99% in the N-responsive DEGs.

The association of different types of G4s with different genomic regions is considered to suggest their role in different regulatory processes. For example, G2 G4s are implicated in the regulation of transcription and translation (Varshney et al., 2020), while G3 G4s are considered important for promoter regulation (Hegyi, 2015). Gene ontology analysis of all the DEGs containing G4s suggests their involvement in carbohydrate metabolism, nitrogen transport, signaling, respiration, and water deprivation among others (**Supplementary Table S17**). These observations are in line with the findings of Yadav et al. (2017), who linked G4s with carbohydrate metabolism and water deprivation. Therefore, it is attractive to validate their role in N-response/NUE, for which we provide a prioritized list of 389 G4s-containing genes common to N-response and grain yield (**Supplementary Table S18**). Further, their regulation needs to be examined in both genetic and epigenetic terms, as G4 sequences are also known to be involved in epigenetic regulation (Cavalieri and Spinelli, 2019; Mukherjee et al., 2019; Reina and Cavalieri, 2020; Wu et al., 2021). Accordingly, the confirmed candidates may be targeted to improve NUE either genetically or epigenetically.

Our RT-qPCR studies validated the nitrate-regulation of seven G4Q harboring genes related to NUE (N-responsive and yield-related) in two Indian rice genotypes (**Supplementary Figure 2**) that we earlier characterized as contrasting for NUE (Sharma et al., 2018; Sharma et al., 2021). Four of these genes showed contrasting patterns of nitrate regulation between the contrasting genotypes, while the other three genes showed similar up/down regulation between genotypes. These differences in gene expression could be due to genetic and/or epigenetic reasons including G4Q and need further investigation using many more genes in wider germplasm. It also remains to be seen whether post-transcriptional regulation of these genes by G4Qs causes measurable changes in their protein levels and whether they correlate with measurable variation of NUE in the germplasm.

Interestingly, G4 sequences can play a potential role in mitochondria (Falabella et al., 2019) and mitochondrial transporters are emerging as an important class of putative G4s-containing candidates in our study. Further, our earlier findings demonstrated the role of mitochondrial respiration in N-response/NUE (Sharma et al., 2018). Taken together, these two lines of evidence indicate the potentially important role of mitochondria in the epigenetic regulation of N-response/NUE, which was so far attributed only to miRNAs. Further, validation of the role of G4s in mitochondrial regulation of NUE could offer novel means to shortlist candidate genes for crop improvement towards NUE.

**Figure 7:**
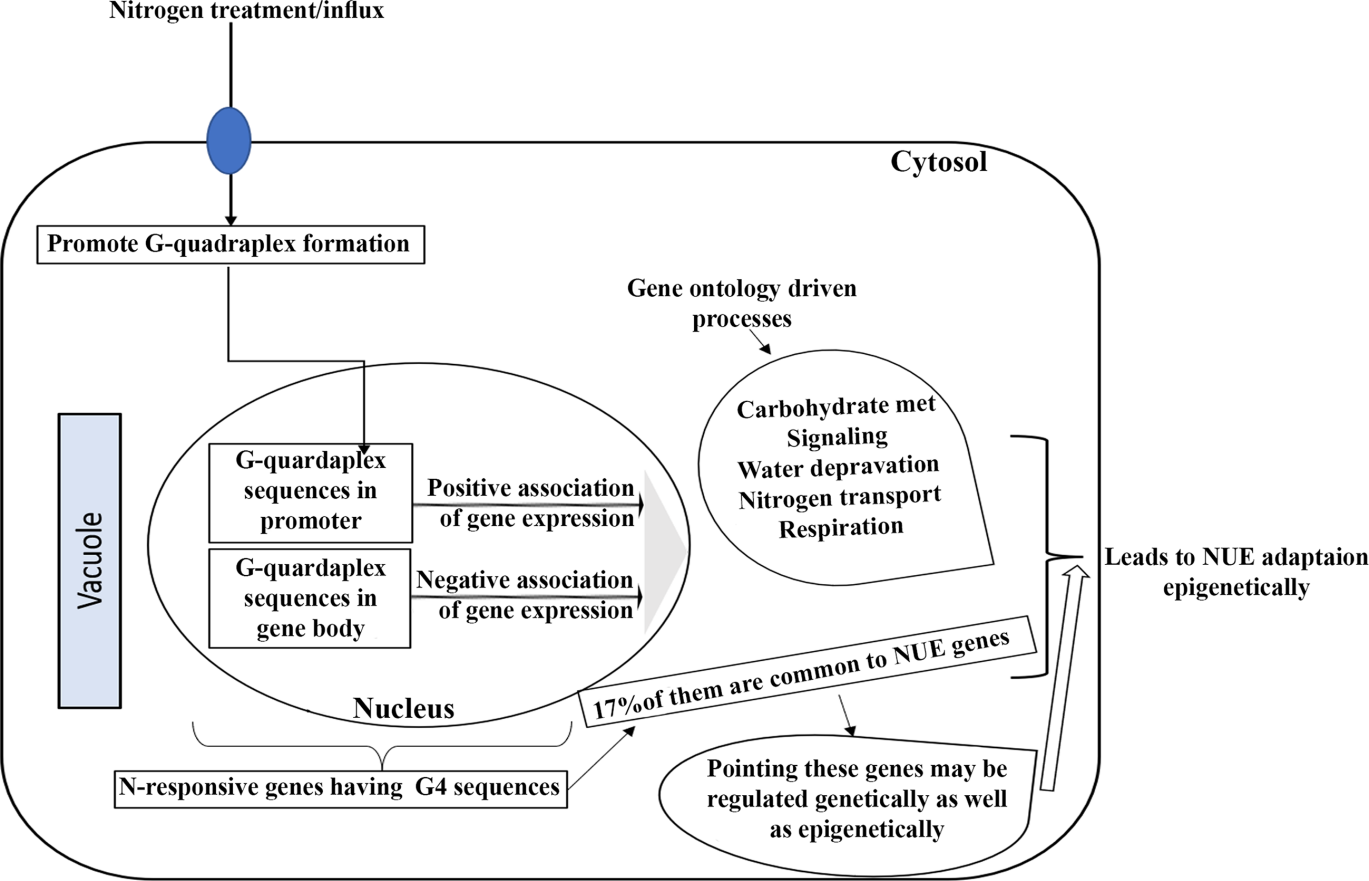
A hypothetical model of the underlying regulatory processes that may be triggered by G-quadruplexes in response to change in the availability of external nitrogen.

Finally, based on our results, we present a model that summarizes the regulatory mechanisms potentially activated by G4 quadruplexes in response to variations in external nitrogen levels (**Figure 7**). Interestingly, only 17% of the G4s-containing N-responsive genes are found to be related to yield, indicating that only those G4s-containing genes that are both N-responsive and yield-related contribute to NUE. These findings add to our previous experimental distinction between N-response at the level of phenotype as well as genotype (Kumari et al., 2021; Sharma et al., 2021; Sharma et al., 2022) and could facilitate the shortlisting of target genes for crop improvement towards NUE as well as to choose between genetic or epigenetic means.

## Data availability statement

The original contributions presented in the study are included in the article/**Supplementary Material**. Further inquiries can be directed to the corresponding author.

## Author contributions

NS performed most of the experiments, analyzed the data, and wrote the first draft. BM performed N-responsive gene-trait association and helped in MS drafting. MSK helped in G-quadruplexes experimental design, data analysis, and MS drafting. KS helped WGCNA analysis. NR helped in the planning, mentoring, and supervision of the experiments, data interpretation, editing, and finalizing of the manuscript. All authors read and approved the submitted version.

## Supplementary material

Supplementary Figure 1 Venn selection representing hierarchical shortlisting of N-responsive DEGs used for our WGCNA study.

Supplementary Figure 2 RT-qPCR graph showing log_2_ fold change values of 7 differentially regulated genes represented as mean± SE in Nidhi and Panvel1 genotypes grown under low-nitrate (1.5 mM) with normal nitrate (15 mM) as control, *OsCYP20-2* (Os05g0103200), *OsGLP2-1* (Os02g0532500), *TubA2* (Os11g0247300), *OsDXS2* (Os06g0142900), *SPDT* (Os06g0143700), *OsASNase2* (Os04g0650700) and *OsNRT1.1A* (Os08g0155400).
