# Supplementary figures and images for: Weighted gene co-expression network analysis of nitrogen (N)-responsive genes and the putative role of G-quadruplexes in N use efficiency (NUE) in rice
Source: Front Plant Sci. 2023 Jun 7;14:1135675. doi: 10.3389/fpls.2023.1135675 (PMC10282765; doi:10.3389/fpls.2023.1135675)

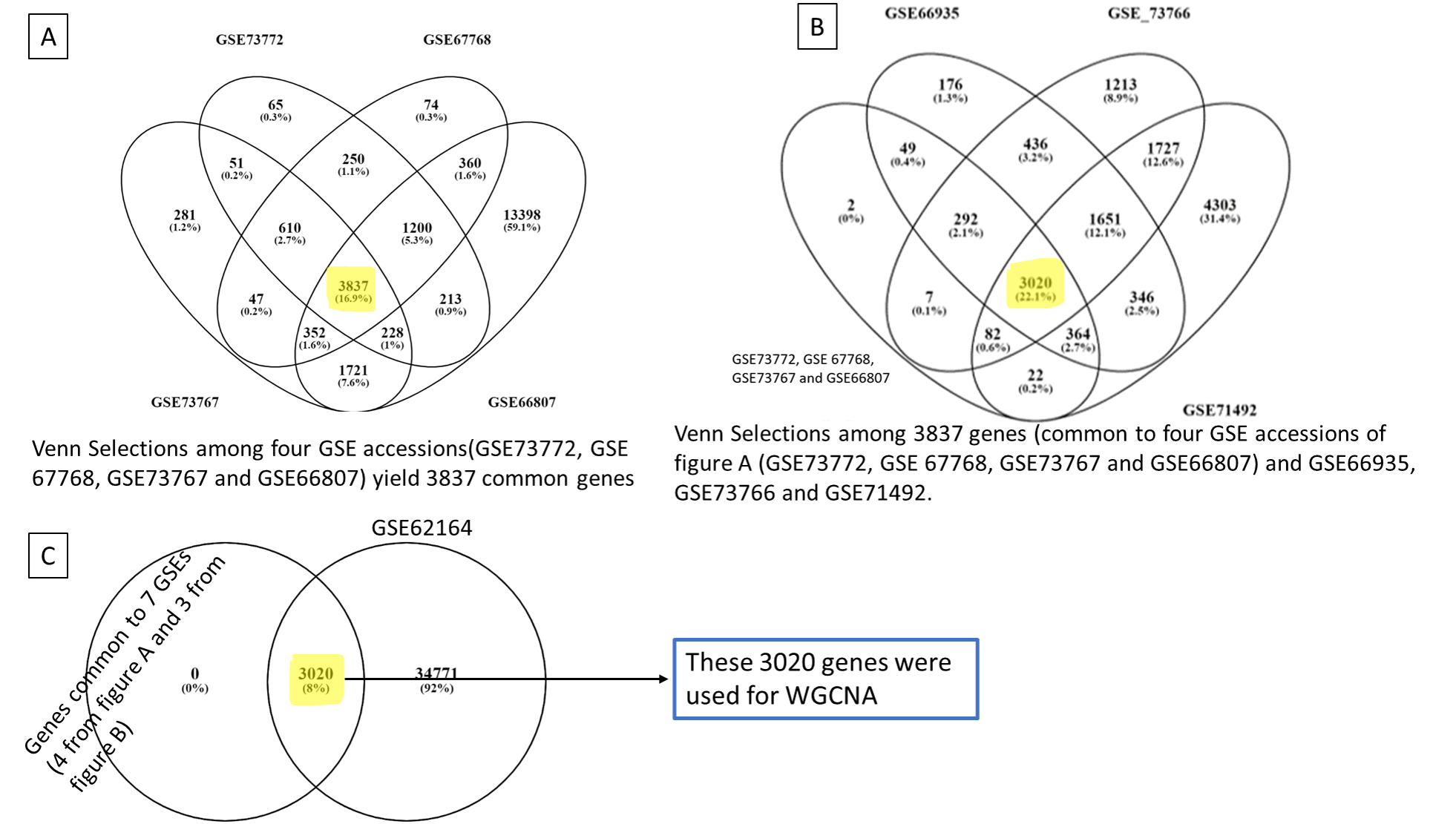

Supplement: Supplementary Figure 1 — Venn selection representing hierarchical shortlisting of N-responsive DEGs used for our WGCNA study. [file Image_1.tif]

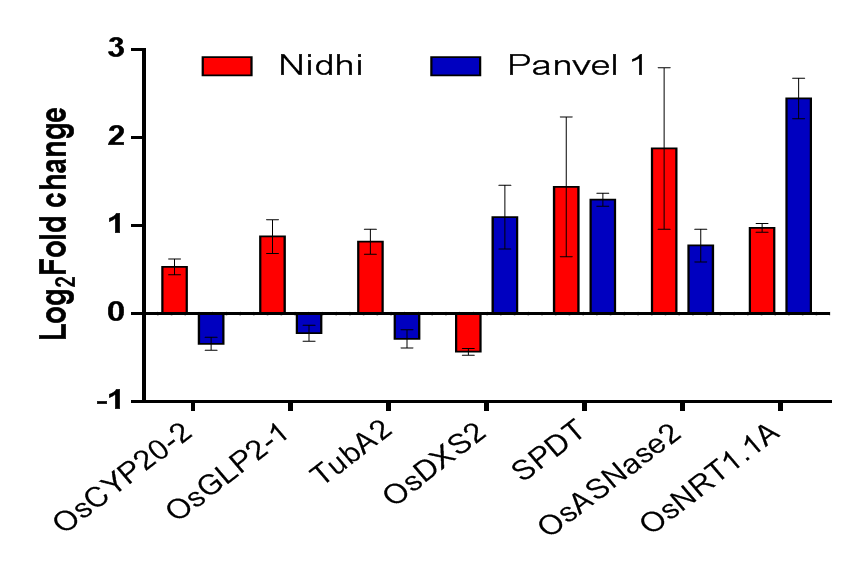

Supplement: Supplementary Figure 2 — RT-qPCR graph showing log2 fold change values of 7 differentially regulated genes represented as mean± SE in Nidhi and Panvel1 genotypes grown under low-nitrate (1.5 mM) with normal nitrate (15 mM) as control, OsCYP20-2 (Os05g0103200), OsGLP2-1 (Os02g0532500), TubA2 (Os11g0247300), OsDXS2 (Os06g0142900), SPDT (Os06g0143700), OsASNase2 (Os04g0650700) and OsNRT1.1A (Os08g0155400). [file Image_2.tif]
